# Malignant transformation of an intracranial epidermoid cyst into squamous cell carcinoma: a case report and systematic review

**DOI:** 10.3389/fonc.2026.1978480

**Published:** 2026-10-28

**Authors:** Lorraine Edna Onzere, Jinan Ma, Elihuruma Eliufoo Stephano, Peter Mutinda Kisangai, Yunfei Li, Weini Quan, Yuanqiang Wu

**Affiliations:** 1 Department of Oncology, The Second Xiangya Hospital, Central South University, Changsha, Hunan, China; 2 Village Market Department, M.P. Shah Hospital, Nairobi, Kenya; 3 Clinical Teaching and Research Section, The Second Xiangya Hospital, Central South University, Changsha, Hunan, China

**Keywords:** cholesteatoma, intracranial epidermoid cyst, malignant transformation, radiotherapy, squamous cell carcinoma

## Abstract

**Objective:**

Intracranial epidermoid cysts (IECs) are rare, benign congenital lesions that occasionally undergo malignant transformation into squamous cell carcinoma (SCC). This study presents a rare case of an IEC in the anterior cranial fossa transforming into SCC and reviews existing literature to summarize clinical characteristics, treatment strategies, and survival outcomes.

**Methods:**

We detail the clinical presentation, management and long-term follow-up of a 52-year-old female who developed SCC 75 months after initial subtotal resection of an anterior cranial fossa IEC. We also conducted a comprehensive systematic literature search across PubMed, MEDLINE and CNKI in accordance with PRISMA guidelines compiling 59 well-documented cases (including our index case) to analyze demographic features, transformation intervals, treatment modalities and overall survival.

**Results:**

Our patient underwent repeat subtotal tumor resection followed by adjuvant intensity-modulated radiation therapy (IMRT). She maintained a disease-free survival (DFS) of 14 months post-radiotherapy (17 months post-reoperation) until July 2026, when she developed multifocal intraventricular recurrence. The literature review revealed a mean patient age of 54.7 years with an equal gender distribution (1.03:1). The cerebellopontine angle (CPA) was the most frequent tumor site (50.85%). The mean interval from IEC diagnosis to malignant transformation was 9.24 years. Prognosis for transformed SCC remains poor, with a median overall survival of 6 months. However, patients treated with surgery combined with radiotherapy demonstrated significantly improved outcomes (median survival 13 months) compared to those undergoing surgery alone (41 days).

**Conclusions:**

Malignant transformation of IECs into SCC is a rare, aggressive complication. Long-term surveillance with contrast-enhanced MRI is vital for patients with previously resected IECs. Early detection, maximal safe surgical resection followed by adjuvant radiotherapy currently offers the most favorable survival benefit.

## Introduction

Intracranial epidermoid cysts (IEC) are rare congenital benign lesions, representing 0.2% to 1.8% of all primary intracranial tumors. Arising from trapped ectodermal elements during embryonic neural tube closure between the 3^rd^ and 5^th^ weeks of gestation, these slow-growing lesions rarely undergo malignant transformation into secondary carcinomas (1). Malignant transformation into SCC is extremely rare, accounting for 0.01% to 0.045% of cases (2).

Since the first reported case in 1912, it has been observed that this malignant degeneration occurs primarily in elderly male patients (3–5). Malignancy can present *de novo* at initial presentation or, even less frequently, years to decades following initial subtotal surgical resection. Once transformed, the clinical course is aggressive (3). The prognosis of malignant transformed IECs is poor with a median overall survival (OS) of only 9–10 months (4–6). Patients with leptomeningeal carcinomatosis (LC) have an even worse prognosis with an OS of approximately 3 months (6). The preferred primary treatment is gross total resection (GTR) (4). Where GTR is unachievable due to critical neurovascular adherence, postoperative adjuvant radiotherapy plays a crucial role in extending survival (3, 5).

Here, we report the case of a 52-year-old female who developed SCC within the original cyst bed 75 months after subtotal resection of an anterior cranial fossa epidermoid cyst. Following repeat surgical resection and adjuvant IMRT, she achieved 14 months of DFS before developing multifocal intraventricular recurrence at 17 months post-operation. Inspired by this case, we conducted a systematic analysis reviewing cases of malignant transformation from IECs into SCC, identifying 97 cases from 1960 to 2026 in literature. Fifty-eight of the ninety-seven cases provided detailed information for analysis. We summarized key details, such as duration from initial diagnosis to malignant transformation, treatment responses and prognostic factors to provide comprehensive suggestions for the diagnosis, treatment and follow-up of this rare transformation.

## Methods

A narrative description of the clinical presentation, neuroimaging, surgical intervention, histopathology, and follow-up of our index case was compiled.

A systematic literature search without publication date restrictions was conducted across PubMed, MEDLINE, and CNKI adhering to Preferred Reporting Items for Systematic Reviews and Meta-Analyses (PRISMA) standards (**Supplementary File 1**). Search strategies combined terms including “intracranial epidermoid cyst” or “brain epidermoid cyst” and “squamous cell carcinoma” or “intracranial squamous cell carcinoma” or “malignant transformation” or “cancer” or “carcinoma” or “malignancy”. Reference lists of retrieved articles were cross referenced to identify additional cases. To ensure methodological rigor, two independent reviewers (L.E.O. and Y.W.) screened the titles, abstracts, and full texts of the retrieved articles for eligibility. Any disagreements between the reviewers were resolved through consensus or by consultation with a third senior reviewer (J.M.). A predefined standardized data extraction form was utilized to systematically collect patient demographics, tumor location, cyst size, time to transformation, surgical extent, adjuvant therapies (radiotherapy dose and fractionation, systemic chemotherapy, or immunotherapy regimens), and overall or disease-free survival. The methodological quality and risk of bias for the included case reports and case series were independently evaluated using the Joanna Briggs Institute (JBI) Critical Appraisal Checklist for Case Reports (**Supplementary File 2**). Studies were categorized based on their proportion of “Yes” responses to the 8-item checklist; those scoring ≥75% (at least 6 of 8 criteria) were classified as high quality with a low risk of bias. Because cases with incomplete clinical datasets were systematically excluded during the initial screening phase, all 58 studies retained for the final synthesis met this high-quality threshold.

### Inclusion criteria

Histopathologically confirmed primary or secondary SCC arising within or adjacent to a documented IEC.

### Exclusion criteria

Cases with unverified initial pathological records, incomplete clinical outcomes, or transformation into non-SCC malignancies (e.g., melanoma, adenocarcinoma).

Time to malignant transformation was defined as the interval between initial histological confirmation of benign IEC and subsequent histological diagnosis of SCC. Survival duration was calculated from the date of SCC diagnosis to death or last recorded follow-up.

Statistical analysis were performed using GraphPad Prism (version 10.3.1, GraphPad Software, Boston, Massachusetts, USA, www.graphpad.com). Survival curves were generated using the Kaplan–Meier method, and statistical differences in survival between groups were evaluated using the log-rank (Mantel-Cox) test. A two-sided standard P value of <0.05 (P < 0.05) was considered statistically significant.

### Illustrative case

A 52-year-old woman presented in November 2018, with a seven-year history of episodic occipital headaches that had intensified over the preceding week and were accompanied by nausea and sleep disturbances. Physical and neurological examinations were non-focal. Cranial non-contrast computed tomography (CT) scan revealed an irregular, mildly hypodense lesion with well-defined borders and multiple peripheral punctate calcifications in the anterior cranial fossa, measuring approximately 60 x 42 mm (**Figures 1A, B**). Further evaluation with an enhanced cranial magnetic resonance imaging (MRI) identified an irregular mass in the anterior cranial fossa, approximately 60 x 59 x 45 mm in size. The lesion exhibited heterogeneous signal intensity on T1-weighted imaging (T1WI) and T2-weighted imaging (T2WI) with predominant hyper-intensity on Fluid-Attenuated Inversion Recovery (FLAIR). Diffusion-Weighted Imaging (DWI) showed mixed low and high signals with restricted diffusion. Post-contrast T1WI demonstrated significant patchy peripheral rim enhancement along the lesion borders with a loss of clear demarcation from the adjacent frontal parenchyma and corpus callosum (**Figures 2A–C**).

**Figure 1:**
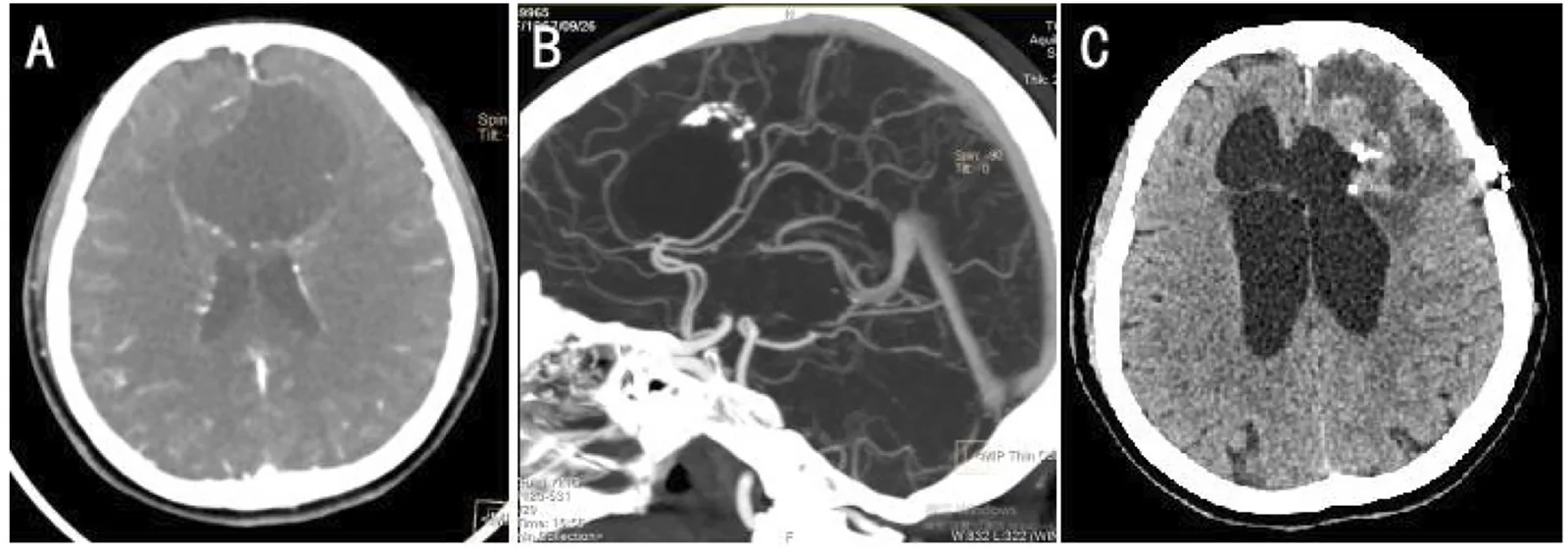
In November 2018, initial head CT demonstrated an irregular, hypodense lesion in the anterior cranial fossa measuring approximately 60×42mm, with multiple punctate hyperdensities along the margin [**(A)** axial view; **(B)** sagittal view]; **(C)** In February 2025, follow-up head CT demonstrated a large, heterogeneous, slightly hypodense mass in the left frontal lobe, containing internal hyperdense foci, alongside a cystic/low-density component in the right frontal lobe communicating with the left lateral ventricle.

**Figure 2:**
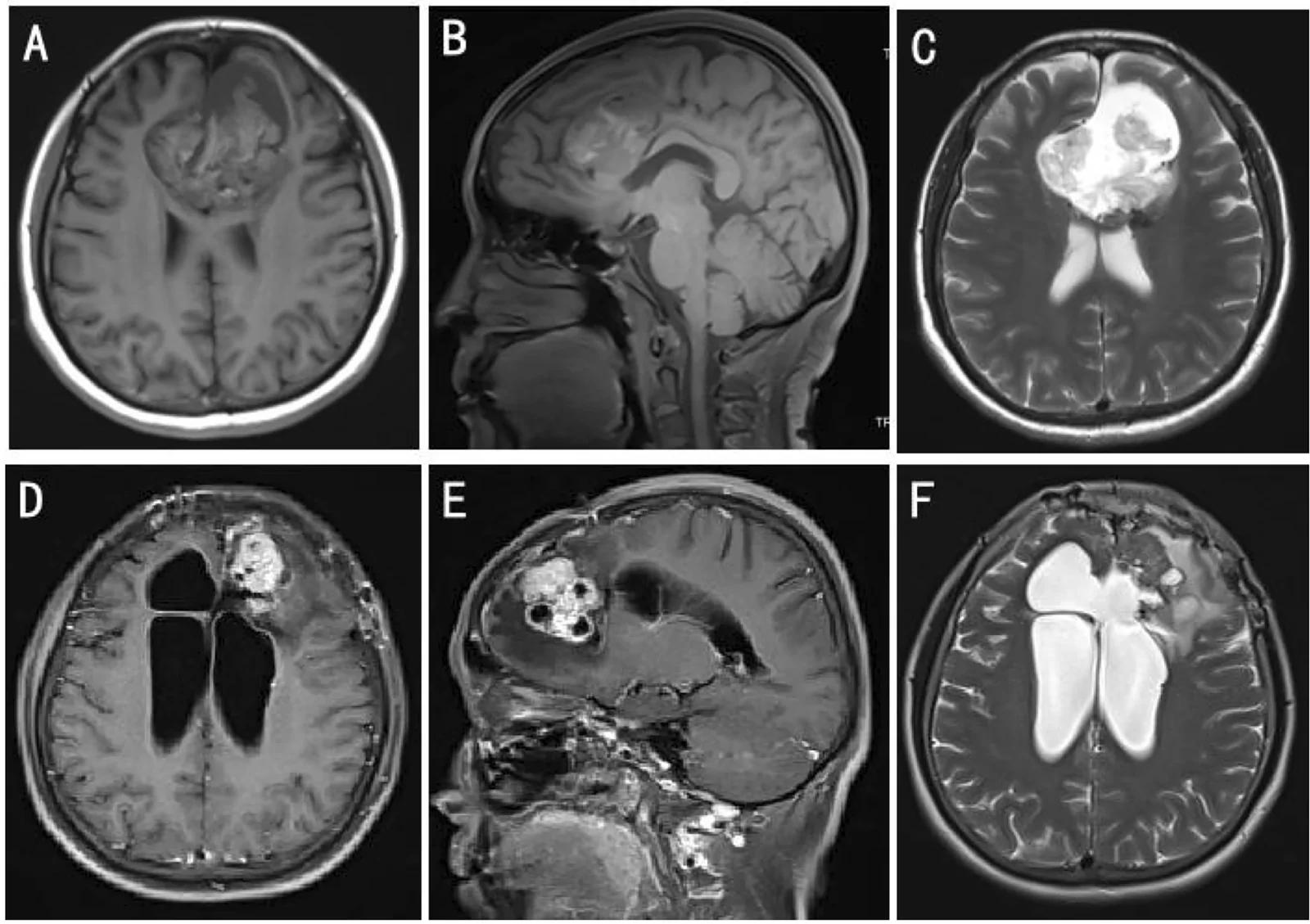
**(A–C)** In November 2018, pre-operative head MRI demonstrated an irregular anterior cranial fossa mass measuring approximately 60×59×45mm with mixed heterogeneous T1 and T2 signal intensity and rim/strip-like contrast enhancement accompanied by surrounding vasogenic edema [**(A)** axial T1-weighted enhanced image; **(B)** sagittal T1-weighted enhanced image; **(C)** axial T2-weighted enhanced image]. **(D–F)** In February 2025, follow-up head MRI revealed a recurrent/ transformed lesion in the left frontal region measuring approximately 42×18mm, displaying mixed isointense-to-T1/T2 signals with prominent ring-like enhancement and adjacent dural enhancement (D. axial T1-weighted enhanced image; **(E)** sagittal T1-weighted enhanced image; **(F)** axial T2-weighted enhanced image.

In November 2018, the patient underwent subtotal surgical resection of the lesion via a frontal craniotomy. The excised lesion measured 65 x 60 x 53 mm and yielded an avascular, pearly-white lesion filled with keratinaceous debris. The cyst capsule adherent to deep frontal cerebral tissue and corpus callosum was left *in situ* to prevent neurological deficit. Histopathological examination confirmed a benign epidermoid cyst (cholesteatoma) (**Figures 3A, B**).

**Figure 3:**
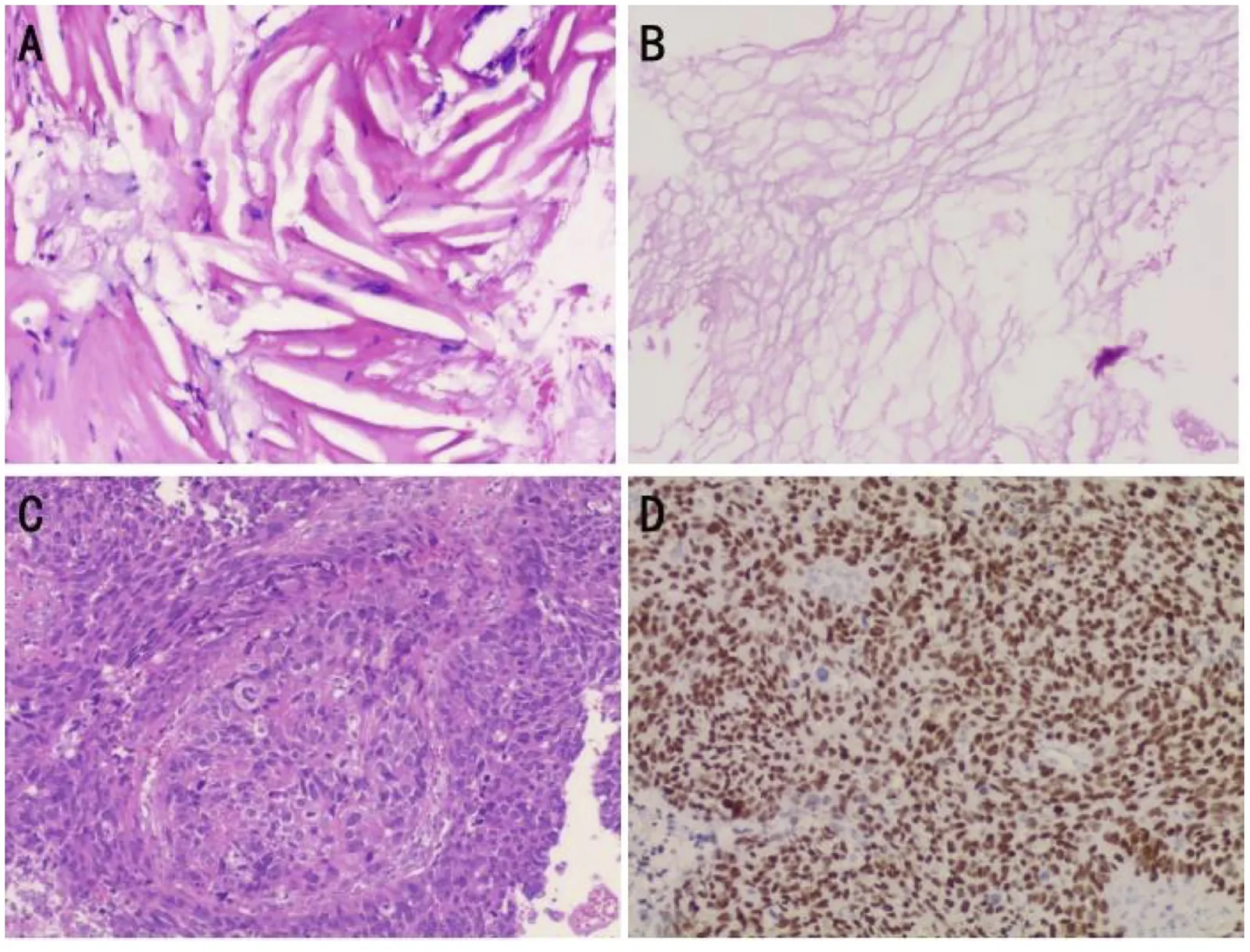
**(A, B)** Pathological examination from the November 2018 resection comfirmed a benign epidermoid cyst (cholesteatoma) in the frontal region [**(A)** Central avascular keratinous debris, H&E x100; **(B)** fibrous cyst wall margin, H&E x100]. **(C, D)** Histopathological analysis from the February 2025 reoperation confirmed malignant transformation to a moderately-to-poorly differentiated squamous cell carcinoma measuring 45x30x20 mm. Immunohistochemical analysis revealed PD-L1 positivity (TPS: 30%; CPS: 30), p40 (+), CK (+), CK5/6 (+), and preserved mismatch repair protein expression [MSH1(+), MSH2(+), MSH6(+), PMS2(+); pMMR status] [**(C)**. tumor nests, H&E x100; **(D)** strong nuclear positivity for p40, x 100].

Six years and three months later (February 2025), she presented with a three-day history of persistent frontal headaches. Her physical and neurological examinations were unremarkable. Laboratory tests were within normal limits. Cranial CT identified a recurrent, heterogeneous hypodense mass in the left frontal lobe containing internal dense calcifications, extending into the anterior horn of the contralateral lateral ventricle (**Figure 1C**).

Cranial MRI showed an irregular mass measuring 42×18 mm with heterogeneous T1/T2 signal, pronounced nodular and ring-like contrast enhancement, surrounding vasogenic edema, and adjacent dural enhancement (**Figures 2D–F**). 18F-FDG PET/CT confirmed intense focal hypermetabolism restricted to the left frontal mass, excluding systemic primary malignancy or extracranial metastases.

In February 2025, the patient underwent repeat left frontal craniotomy and STR due to extensive involvement of the left frontal lobe and adjacent ventricular wall. An intraoperative cerebrospinal fluid (CSF) leak was successfully repaired. Histopathological evaluation of the resected tissue (45×30×20 mm) revealed moderately to poorly differentiated squamous cell carcinoma with severe nuclear atypia and frequent mitotic figures (**Figures 3C, D**).

Immunohistochemistry showed positivity for P40, CK, focal CK5/6, pMMR status (MLH1+, MSH2+, MSH6+, PMS2+) and a PDL-1 TPS of 30% and CPS of 30. From April to May 2025, she received adjuvant IMRT. The prescribed doses were 66 Gy in 31 fractions to the planning gross target volume (PGTV), 60 Gy to PTV1, and 56 Gy to PTV2. Routine follow-up MRI in January 2026 (11 months post-RT, 14 months post-reoperation) showed postoperative cavity changes with no evidence of residual tumor or progression.

In July 2026, (17 months post-re-operation) the patient developed progressive dizziness, nuchal rigidity and cognitive decline. Contrast-enhanced cranial MRI demonstrated multiple new intraventricular enhancing nodules (up to 16 x 12mm) and progressive supratentorial hydrocephalus, consistent with multi-focal ventricular recurrence and leptomeningeal dissemination. Re-operation was deemed unfeasible due to diffuse disease and palliative systemic therapy with bevacizumab and temozolomide was initiated. Following evaluation for re-irradiation, the patient commenced intensity-modulated radiation therapy (IMRT) late July 2026. A subsequent follow-up contrast-enhanced cranial MRI in August confirmed the new lesions were sensitive to the radiotherapy, revealing a size reduction of the intraventricular enhancing nodules, with the largest lesion decreasing from 16 × 12 mm to 11 × 8 mm.

### Literature review results

Our literature search identified 97 published cases of malignant transformation of IEC into SCC. After excluding cases with incomplete clinical datasets or unverified initial histology, 58 published cases met all the criteria (**Figure 4**). Combined with our index patient, the final analytical cohort comprised 59 patients (**Tables 1**, **2**). Quality assessment utilizing the JBI Critical Appraisal Checklist indicated that the 58 included cases possessed sufficient methodological quality, with adequate clinical history and histopathological confirmation (**Supplementary File 2**).

**Figure 4:**
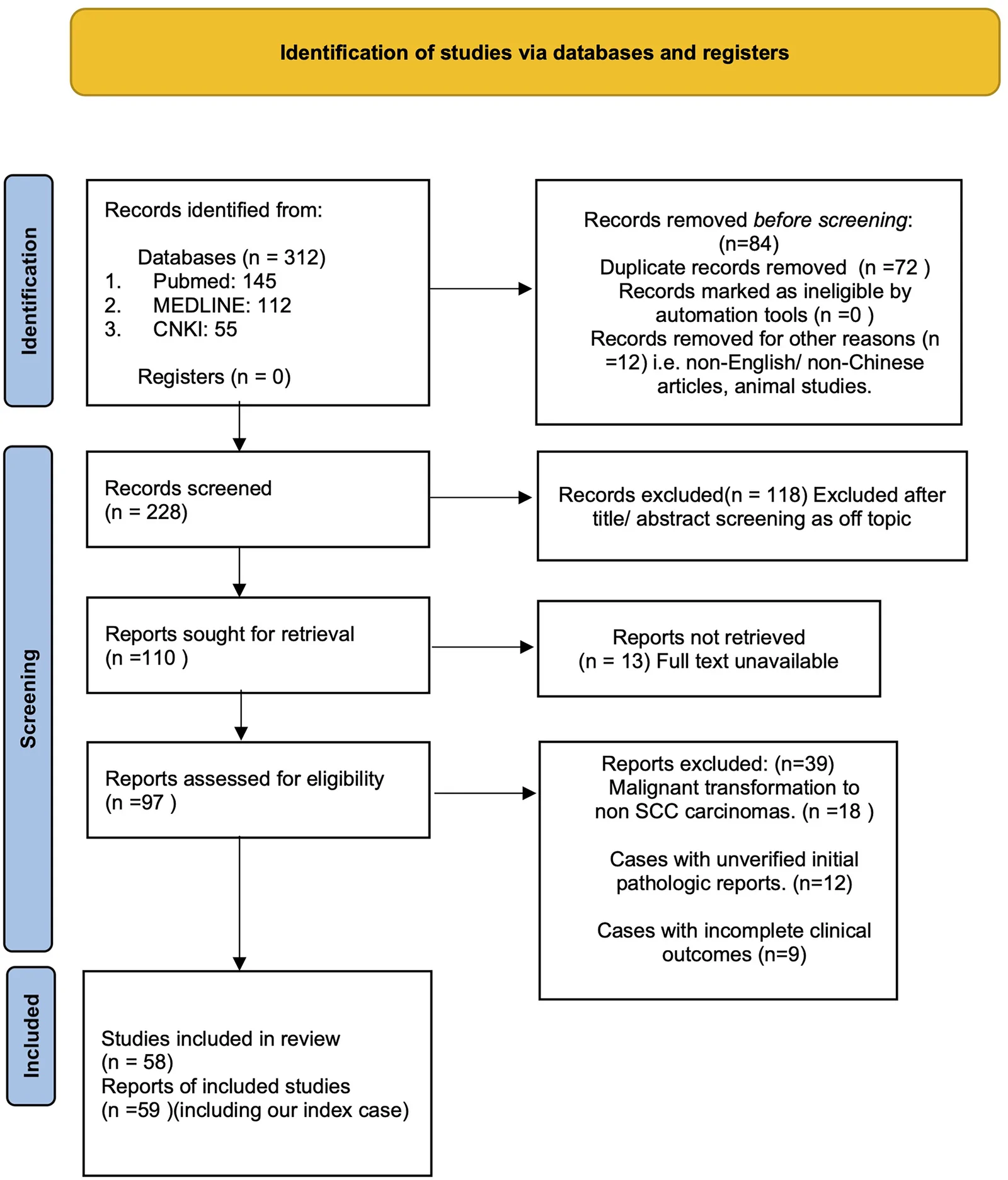
PRISMA flow diagram summarizing the literature search and study selection process. PRISMA, Preferred Reporting Items for Systematic Reviews and Meta-Analyses.

**Table 1:** The principal characteristics of patients with intracranial SCC transformed from IEC included in previous report and in our study.

Reference	Gender	Age	Location	Cyst Size	Time of transformation	Treatment	Radiotherapy dose and frequency	Medical therapy	Overall survival/ follow-up time
Davidson (1960) (29)	M	46	Frontal	Not specified	Not specified	GTR	Not specified	No	Not specified
Fox H et al., (1965) (30)	M	43	Anterior Temporal	Not specified	7 years	STR	None	No	6 weeks
Toglia (1965) (31)	F	53	Base of brain	Not specified	12 months	STR	None	No	1 day
Salyer (1973) (32)	F	58	Pituitary	0.25cm width	Not specified	GTR +RT	Not specified	No	5 weeks
Garcia (1981) (33)	M	61	CPA	4.1x2.7cm	Not specified	GTR+RT	Not specified	No	49 months
Lewis (1983) (34)	F	54	Parasellar	Not specified	Not specified	STR	None	No	1 month
Bondeson (1984) (35)	F	56	CPA	Not specified	Not specified	Medical	None	No	0.75 months
Goldman S et al., 1987 (36)	F	59	Intraventricular	Not specified	33 years	STR+ RT	5000 rads bitemporal radiation therapy	Not specified	Alive at follow-up of 36 months
Abramson et al., 1989 (37)	M	36	CPA	Not specified	2 years	STR	Not specified	Not specified	Not specified
Knorr (1991) (38)	M	74	CPA	3cm	13 months	STR+RT	50.4 Gy	No	7 weeks
Delangre et al., (1992) (39)	F	72	Left CPA	Not specified	Initial diagnosis	STR	Not specified	No	Not specified
Kobayashi et al., 1993 (23)	F	59	Right suprasellar	Not specified	20 years of epilepsy before diagnosis	STR+RT+ Chemotherapy	40 Gy in 30 fractions	Bleomycin (stopped at 105mg due to liver dysfunction)	6 months
Acciarri et al., (1993) (40)	M	62	Parasellar	Not specified	Initial diagnosis	STR	Not specified	No	10 days
Nishio et al., (1995) (41)	M	57	CPA	Not specified	Not specified	STR+RT	Local irradiation of 50 Gy	Not specified	Alive at follow-up of 2.5 years
Nishio et al., (1995) (41)	M	42	Right middle fossa	Not specified	9 years	STR	Not specified	Not specified	Not specified
Fuse et al., (1995) (42)	F	74	Para pontine	Not specified	Initial diagnosis	S+RT	Not specified	No	12 months
Bayindir et al., (1996) (43)	F	67	Intraventricular	Not specified	Not specified	S	Not specified	Not specified	Alive at follow-up of 26 months
Murase et al., 1999 (24)	F	50	Right CPA	Not specified	10 years	STR+RT+Chemotherapy	Gamma radiosurgery, marginal dose 14 Gy	VMP-F regimen	Alive at follow-up of 5 years
Sawan et al., (2000) (44)	M	66	Prepontine	3cm x 1cm	At autopsy	Shunt	Not specified	Not specified	6 months
Tsugu et al., 2001 (45)	M	47	Cerebellar vermis left cerebellar hemisphere+ cisterna magna	Not specified	Not reported	GTR+RT	55 Gy local radiotherapy	No	Alive at follow-up of 6 years with no recurrence
Asahi et al., (2001) (46)	F	55	CPA	Not specified	13 years	S	Not specified	Not specified	3 months
Nawashiro et al., (2001) (47)	M	46	Temporal	Not specified	Not specified	S	Not specified	Not specified	Not specified
Khan et al., (2001) (48)	M	53	Prepontine	Not specified	9 months	Shunt	No	No	9 months
Link et al., (2002) (49)	F	57	CPA	Not specified	1 year	STR+RT	External beam: 45 Gy in 28 fractions; Stereotactic boost: 15Gy	No	3 years
Monaco et al., (2003) (50)	M	36	Posterior fossa	Not specified	Not specified	S	Not specified	Not specified	Alive at follow-up of 2 years
Hamlat et al.,(2004) (2003) (51)	F	62	Temporal	Not specified	Not specified	Chemotherapy	Not specified	Not specified	7 months
Michael L et al., 2005 (22)	M	45	Posterior Fossa	Not specified	At recurrence (CSF leakage)	STR+RT+ Chemotherapy	59.4 Gy in 33 fractions	Cisplatin+5-fluorouracil	12 months
Tamura K et al., 2006 (52)	F	56	Left CPA	Not specified	8 years	STR+Stereotactic radiosurgery	2 Cycles: 15Gy-25Gy and 12Gy-20Gy	Not specified	Alive at follow-up of 18 months
Ge P et al., 2009 (53)	M	50	Right Temporal Lobe	Not specified	6 years	GTR+RT+ chemotherapy	Not specified	Not specified	Not specified
Kano T et al., 2010 (54)	F	64	Medial Temporal Region	3.5x2.5cm	16 years	STR + RT	Local 50 Gy	Not specified	2 years
Nakao Y et al., 2010 (8)	F	74	Right CPA	Not specified	20 years	STR + RT	46 Gy linac radiotherapy	Not specified	Alive at follow-up of 17 months
Hao S et al., 2010 (55)	F	61	Right CPA, Pre-pontine cistern and Temporoparietal lobe	Not specified	6 years	STR	No	No	36 days
Lakhdar F et al 2011 (56)	M	52	CPA	Not specified	6 months	GTR + RT	Local Radiotherapy of 50Gy	Not specified	Alive at follow-up of 1 month
Chon K et al., 2012 (10)	M	43	CPA	5.8x3.8cm	2 years	STR+ Gamma Knife Radiosurgery	15-25 Gy	Not specified	Not specified
Vellutini E et al., 2014 (57)	F	42	Right CPA	Not specified	2 years	STR	None reported	No	40 days
Pikis S et al., 2016 (58)	M	77	Left CPA	Not specified	9 months	STR + RT	Local RT of 55Gy	Declined chemotherapy	6 months
Ding S et al., 2016 (59)	F	55	Left Temporal Region and Prepontine area	Not specified	7 months	STR	Declined RT	No	6 months
Seif B et al., 2017 (60)	M	83	Posterior Fossa	Not specified	2 months	GTR	Not specified	Not specified	Not specified
Mascarenhas A et al. (2017) (11)	M	20	Midline posterior fossa	Not specified	Detected at first surgery	Surgical resection (Near total) + RT	Not reported	No	13 months
Roh et al (2017) (61)	F	53	CPA	Not specified	At initial diagnosis	GTR+RT	67.2Gy in 28 fractions	No	4years
Liu et al., (2018) (62)	M	20	Left lateral ventricle	3.6×4.3×3.5 cm	At initial diagnosis	S	Not specified	No	9 months
Cuoco J et al 2019 (16)	M	71	Left CPA	Not specified	4 decades	STR + stereotactic radiosurgery	25 Gy in 5 fractions	No	Not specified
Kwon SM et al., 2019 (63)	M	57	CPA	Not specified	3 years	GTR+RT	Not specified	No	48 months
Kwon SM et al., 2019 (63)	F	61	CPA	Not specified	11 years	STR+RT	Not specified	No	25 months
Chen et al., (2019) (18)	M	43	CPA	Not specified	Primary	Proton beam therapy	57 Gy in 31 fractions	Not specified	Healthy and functional at follow-up of 2 years
Faltaous et al., (2019) (64)	M	77	Left parietal	2.1x2.1cm	Not specified	S	No	No	Not specified
Zuo P et al., 2021 (3)	M	61	Right CPA	Not specified	At initial diagnosis	STR	No	No	12 months
Narasimhaiah et al. (2022) (65)	M	39	CPA	2.3x1.6x2.9cm	5 months	GTR	Not specified	Not specified	Died shortly after recurrence
Sakamoto et al., (2022) (66)	F	59	CPA	Not specified	8 years	STR + stereotactic radiotherapy	50Gy in 25 fractions	Not specified	Alive at follow-up of 7 months without recurrence
Eatz T et al., 2023 (5)	F	43	CPA	4.9x2.6x2.9cm	25 years	STR+RT	59.4Gy in 33 fractions external beam radiation	No	Not specified
Zhang et al., (2023) (67)	M	58	Right frontoparietal lobe	Not specified	At initial diagnosis	S + RT	6000 cGY in 30 fractions	No	Alive at follow-up of 1 year
Jeon J et al., 2024 (68)	F	56	Right CPA and Meckel’s Cave	4.3cm	At initial diagnosis	S + chemotherapy (halted due to adverse effects)	Not specified	Chemotherapy used, drug not specified	2 months
Song et al., (2024) (28)	F	47	Left internal capsule	Not specified	At initial diagnosis	S + Chemotherapy+ Targeted therapy +RT	Not specified	Chemotherapy used, drug not specified	Effectively controlled at follow-up (timeline not specified)
Lin & Ding (2024) (69)	F	55	CPA	Not specified	At initial diagnosis	STR	Declined RT	No	Recurrence at follow-up of 1year
Gasca Saldaña et al., 2025 (19)	F	55	Right CPA	Not specified	25 years	STR (>90% removed) +RT+ Chemotherapy+ immunotherapy	30 Gy in 5 fractions	Carboplatin+ paclitaxel+ pembrolizumab	Alive at follow-up of > 2 years
Alsadi et al., (2025) (20)	M	40	CPA	Not specified	14 years	Shunt + chemotherapy+ immunotherapy	None	Paclitaxel+ carboplatin +cemiplimab	2 months
Elsarraj & Hogan (2025) (15)	M	61	CPA	3.1x2.1cm	14 years	STR+ chemotherapy + immunotherapy	Not specified	Carboplatin +paclitaxel+ cemiplimab	2 months
Tsai C et al., 2026 (21)	F	48	Left CPA	Not specified	14 months	STR + Adjuvant Chemotherapy+ RT	26.7 Gy in 10 fractions	Pembrolizumab +carboplatin, later capecitabine	Alive at follow-up of 20 months
CURRENT CASE PRESENTED	F	58	Anterior cranial fossa	6.0x5.9x4.5cm	6 years and 3 months	STR + RT+ chemotherapy + immunotherapy	59.4Gy in 33 fractions	Temozolomide + Bevacizumab	Intraventricular recurrence at 14 months post-RT (17 months post-reoperation, July 2026)

SCC, Squamous Cell Carcinoma; IEC, Intracranial epidermoid cyst; M, Male; F, Female; CPA, Cerebellopontine Angle; S, Surgery; RT, Radiotherapy; GTR, Gross Total Resection; STR, Sub-Total Resection; VMP-F, Vincristine sulfate, Methotrexate, Peplomycin, Calcium folinate.

**Table 2:** Summarized characteristics of patients with epidermoid cyst to squamous cell carcinoma transformation.

Characteristic	Number of cases	Proportion /%	Mean interval time	Median follow-up time	Median survival time
Gender					
Male	30	50.85%	(15 cases) 8.15 years	(22 cases) 12.00 months	(14 cases) 7.50 months
Female	29	49.15%	(17 cases) 12.94 years	(26 cases) 12.00 months	(16 cases) 6.00 months
Location					
Cerebellopontine angle	30	50.85%	10.15 years	14.50 months	10.45months
Temporal/middle fossa	8	13.56%	7.00 years	30.00 months	14.50months
Cerebellar/vermis/posterior fossa	5	8.47%	0.00 years	41.00 months	7.00 months
Sellar/parasellar/pituitary/base of brain	5	8.47%	1.00 year	–	1.25 months
Brainstem/prepontine/Para pontine	4	6.78%	0.00 years	–	6.00 months
Ventricular/internal capsule	3	5.08%	33.00 years	26.00 months	9.00 months
Occipital/parietal/frontoparietal	3	5.08%	0.00 years	12.00 months	–
Frontal lobe/Anterior cranial fossa	1	1.69%	6.25 years	17.00 months	–
Time from IEC to SCC					
≤1 year	8	25.00%	0.65 years	(6 cases) 6.00 month	(5 cases) 6.00 months
1-2 years	5	15.63%	1.79 years	(2 cases) 10.50 months	(2 cases) 44.5 days
2-5 years	1	3.12%	3.00 years	(1 case) 48.00 months	(1 case) 48.00months
>5 years	18	56.25%	15.51years	(15 cases) 17.00 months	(8 cases) 2.5months
At initial diagnosis/ Autopsy/Untracked	13	–	–	(11 cases) 12.00 months	(8 cases) 12.00months
Time from SCC to death					
≤6 months	18	58.06%	10.34 years	–	6.5 weeks
6-12 months	6	19.35%	3.20 years	–	10.5 months
12-24 months	2	6.45%	6.75 years	–	18.5 months
>24 months	5	16.13%	20.40 years	–	48 months
Not reported (Alive/stable/recurrence)	28	–	10.22 years	22 months	–

IEC, intracranial epidermoid cyst; SCC, squamous cell carcinoma.

The patient age ranged from 30 to 83 years (mean 54.7 years). The cohort included 30 males (50.85%) and 29 females (49.15%) (male to female ratio 1.03: 1). The most frequent tumor location was the cerebellopontine angle (CPA, n=30, 50.85%), followed by the temporal/middle cranial fossa (n=8, 13.56%), cerebellar/vermis/posterior fossa (n=5, 8.47%), sellar/parasellar/pituitary/base of brain (n=5, 8.47%), brainstem/prepontine/parapontine region (n=4, 6.78%), intraventricular/internal capsule (n=3, 5.08%), occipital/parietal/frontoparietal region (n=3, 5.08%), and the frontal lobe/anterior cranial fossa (n=1, 1.69%). Most cases did not mention the cyst size. Among the 13 cases with documented sizes, the maximum diameter ranged from 0.25 to 6cm, with an average of 3.6 cm. Our index case represented the largest tumor size (**Tables 1**, **2**).

Thirty-two cases reported the exact time interval from benign IEC resection to SCC transformation. The mean transformation interval was 9.24 years (range: 2 months to 40 years; median 6.1 years). Over 56% (56.25%) of patients developed malignant transformation after 5 years.

Forty-six cases provided exact survival follow-up. Survival following SCC diagnosis ranged from 1 day to 6 years, with a median overall survival of 1 year. Over half (31/59, 52.5%) of the patients were reported dead on follow-up, with a median survival time of 6 months (range: 1 day to 49 months). Only two patients survived beyond 5 years (**Tables 1**, **2**).

53 of 59 patients (89.8%) underwent surgical intervention for the malignant lesion. GTR was achieved in 10 cases (18.9%), STR in 33 cases (62.3%). Surgical extent was unspecified in 10 cases.

32 of 59 patients (54.2%) received radiotherapy. Of these, 31 patients (96.9%) received adjuvant RT combined with surgery, while 1 patient received definitive proton beam therapy alone.

12 of 59 patients (20.3%) received systematic agents, including 5-fluorouracil, platinum-based chemotherapy (n=11) (91.7%) and immunotherapy (n=3) (25.0%). 9 of the 12 (75%) received systematic therapy combined with surgery, while 3 patients (25%) received medical management alone (**Tables 1**, **2**).

Patients treated with combined surgery and radiotherapy (n = 13 with specific survival data) achieved a median survival of 13 months, compared to just 41 days for patients treated with surgery alone *n* = 10 (P < 0.01).

## Discussion

Malignant transformation of an intracranial epidermoid cyst is a rare, life-threatening clinical event. Published literature demonstrates that the mean age at malignant transformation ranges from 51.2 to 55.2 years old, with a slight male predominance (3–5). These lesions predominantly arise in the posterior cranial fossa (4, 5, 7). Our patient initially presented at 52 years of age with a benign IEC that progressed to SCC by age 58. Uniquely, her tumor was localized to the anterior cranial fossa, a site reported in only seven previous cases (7.5%) (5). In our systematic review of 59 reported cases, our index case is the sole documented instance in which both the primary IEC and subsequent SCC originated in the anterior cranial fossa. Dural or leptomeningeal involvement, present in our patient, is documented in only 22 patients (16.2% of cases) (5, 6, 8, 9). The wide variation in clinical outcomes among patients with malignant transformation is heavily dependent on anatomical location and tumor resectability. In standard clinical presentations, IECs are located in the cerebellopontine angle, where their dense adherence to the brainstem and critical cranial nerves severely limits the feasibility of a gross total resection (GTR). In contrast, the anterior cranial fossa localization in our index case provided an anatomically permissive environment that, although still resulting in a subtotal resection (STR), allowed for a safe and extensive cytoreduction. This location, which is relatively free from immediate brainstem encasement, also allowed for effective targeted adjuvant radiotherapy without the rigid dose constraints typical of the CPA. These favorable anatomical factors might have directly contributed to our patient achieving a disease-free survival of 17 months post-reoperation, markedly outperforming the historical median survival of 13 months for combined therapy.

The cellular mechanisms driving malignant degeneration of intracranial epidermoid cysts remain unclear. Two major hypotheses exist. The first suggests that the squamous epithelium of the cyst undergoes progressive atypical hyperplasia, where prolonged cell proliferation ultimately leads to malignant transformation (10). The second suggests that chronic inflammatory responses - triggered either by cyst content leakage or surgical manipulation of the residual cyst wall, induce molecular alterations, such as epithelial dysphosphorylation, thereby leading to malignancy (11). Hamlat et al. categorized primary intracranial SCCs into five distinct types: Direct malignant transformation of an initial benign cyst (Type I), Malignancy arising from a post-surgical residual cyst(Type II), Malignant transformation within dermoid or epidermoid cysts without prior intervention (Type III), Malignancy with dissemination of the leptomeninges (Type IV), and Other malignancies arising from benign cysts (Type V) (6). Given our patient’s history of prior STR in 2018 and the subsequent leptomeningeal involvement, her disease exhibits characteristics of both Type II and Type IV classifications.

Clinically, the symptoms and signs of malignant transformation tend to be asymptomatic or nonspecific depending mainly on the cyst’s location and size (4, 7). They include progressive headaches, convulsions, or focal neurological deficits, similar to symptoms of other intracranial space-occupying lesions (12). Early recognition of malignant transformation is therefore challenging. Case in point: Our patient complained of severe headache, nausea, and sleep disturbances, consistent with an intracranial mass rather than an overt malignancy. While clinical presentations often mimic the mass effect of benign cysts, specific indicators can signal malignant transformation. Key red-flag features that should raise suspicion for malignant degeneration include: history of prior resections, particularly subtotal resection, which may lead to chronic inflammation and keratin spillage, thereby increasing the risk of malignant transformation. Additionally, rapid growth of a previously stable cyst, accompanied by sudden increase in neurological symptoms or cerebrospinal fluid leakage, should raise immediate concern. The presence of nodular or ring-like contrast enhancement on CT or MRI, rapid growth, perilesional vasogenic edema, irregular borders, or parenchymal invasion are critical radiological indicators of potential malignancy (3, 4). (Benign IECs typically display well-defined, non-enhancing lesions, low density CT features, T1 hypointensity, T2 hyperintensity, and restricted diffusion on Diffusion-Weighted Imaging (DWI) (13). Elevated tumor markers like serum Carbohydrate Antigen 19-9 (CA 19-9) may further aid in diagnosis and follow-up (14). Cerebrospinal fluid (CSF) test with lymphocytic pleocytosis, elevated protein, and hypoglycorrhachia should alert clinicians to leptomeningeal involvement (9). Histopathological evaluation remains the definitive diagnostic gold standard. Where re-operation is contraindicated, CSF cytological examination can provide diagnostic confirmation (15). Our patient developed SCC after initial STR, which underscores the significance of prior surgical intervention as a risk factor. The initial identification of malignancy was crucially based on MRI findings of patchy enhancement and perilesional edema, which were subsequently confirmed through surgery and histopathology examination.

The timeline for malignant transformation from an IEC to SCC varies significantly. A previous meta-analysis published in 2026 by Saito et al. pooling 103 cases reported a mean interval of 106.1 months from initial diagnosis to malignant transformation, with subgroup intervals of 121 months for patients with recurrent disease (IEC with SCC) and 60 months for primary SCC cases (4). Similarly, another 2021 review by Zuo et al. analyzing 54 cases demonstrated a mean transformation interval of 9.9 years (3), whereas Eatz et al., 2023 reported a substantially shorter median interval of 60 months (5). In our patient, the latency period was 6 years and 3 months (75 months), aligning closely with the broader literature range of 5 to 25 years (5). Across the 59 cases analyzed in **Table 1**, transformation intervals ranged from rapid disease progression at 2 months to delayed transformation up to 48 years yielding a mean interval of 9.24 years (16). Notably, the true onset of malignant degeneration in our case may have occurred earlier, as the patient remained asymptomatic and contrast-enhanced MRI was not performed in a timely manner. Consequently, routine clinical surveillance utilizing contrast-enhanced MRI is imperative for patients with a history of IEC.

Maximum safe GTR provides the best outcome. However, dense capsular adhesions to critical neurovascular structures limits GTR rates to only 18.9% in pooled series. Residual microscopic or macroscopic disease carries a high risk of rapid recurrence. In such cases, adjuvant radiotherapy with or without chemotherapy improves overall survival (4, 17). A pooled cohort of 46 cases analyzed by Zuo et al., 2021 demonstrated that postoperative radiotherapy following GTR yielded a median survival time of 24 months and a 1-year overall survival rate of 72.5%, significantly outperforming the 4 months and 18.2% observed in patients who did not receive radiotherapy (3). Consequently, Zuo et al. concluded that adjuvant radiotherapy significantly improved survival outcomes (3). In our pooled analysis of 59 cases, patients treated with combined surgery and radiotherapy (n=13) achieved a median survival of 13 months, compared to only 41 days for those managed with surgery alone (n=10). Thus, the survival benefit conferred by adjuvant radiation therapy is well established. Furthermore, proton beam therapy, an emerging radiation modality, was demonstrated to be well tolerated over a 2-year follow-up period (18). In the present case, adjuvant radiotherapy was administered following subtotal surgical resection to mitigate the risk of local recurrence or secondary malignant progression. Among the 23 cases detailing radiation parameters, 5 patients received large-dose fractions, while the remaining 18 cases were given conventional fractions with total dose ranging from 39.4 to 67.2Gy (mean: 52.4Gy). Despite this wide dosing spectrum, no clinical consensus on an optimal radiotherapy protocol has been reached. This lack of standardization is inherently driven by anatomy rather than tumor radiobiology; prescribed doses are strictly dictated and limited by the radiation tolerance of adjacent critical central nervous system (CNS) structures, such as the brainstem, optic chiasm, and cranial nerves. As a result, establishing a definitive dose-response relationship in the literature is highly complicated by anatomical heterogeneity, historically small case cohorts, and the evolution of radiotherapeutic techniques over the past several decades.

The therapeutic efficacy of systemic chemotherapy remains unproven due to limited and heterogeneous data. Nevertheless, individual cases demonstrated favorable treatment responses (19). Reported chemotherapeutic regimens in the literature include carboplatin, paclitaxel (15, 19, 20), capecitabine (21) or 5-fluorouracil (22), bleomycin (23), cisplatin (22), and the VMP-F regimen (vincristine, methotrexate, peplomycin, calcium folinate) (24). However, because relatively few published cases report explicit survival time - comprising only 3 patients treated with medical therapy alone and 4 patients managed with combined surgery and medical therapy- calculating a median survival time is unfeasible. Consequently, whether systemic chemotherapy confers a definitive survival benefit remains unclear.

In an effort to explore new therapeutic methods, immune checkpoint inhibitors (ICIs) have recently been used. One case demonstrated good treatment tolerance and prolonged clinical stability following ICI therapy (19). However, two other reported cases, chemo-immunotherapy consisting of carboplatin, paclitaxel, and cemiplimab yielded a survival time of only 2 months (15, 20). Another patient received seven cycles of adjuvant pembrolizumab following surgery and radiotherapy, achieving a 5-month DFS prior to disease recurrence (21). As highlighted by these clinical outcomes, SCC arising from an intracranial epidermoid cyst exhibits a distinct biological behavior from classical head and neck SCC (HNSCC) regarding its sensitivity to radiotherapy, chemotherapy and immunotherapy. The reduced efficacy of modern systemic agents and immunotherapies in this context can be mechanistically attributed to several factors unique to the central nervous system. First, the blood-brain barrier (BBB) severely limits the penetrance of large molecular targeted therapies and systemic cytotoxic drugs into the tumor microenvironment. Second, the CNS immune microenvironment is intrinsically immunosuppressive and isolated. Unlike conventional mucosal HNSCCs, which are highly vascularized and accessible to systemic immunity, intracranial transformed SCCs likely lack the necessary influx of robust effector T-cell infiltration. Without adequate T-cell trafficking, immune checkpoint inhibitors frequently fail to mount a clinically meaningful anti-tumor response, even in the presence of otherwise favorable tumor PD-L1 expression.

The malignant transformation of the cyst epithelium into SCC generally carries a poor prognosis (25). A 2026 review of 103 cases showed that malignant transformation of ECs results in an overall survival time ranging from 1 day to 60 months (4). Another review analyzing 135 cases reported that the mean survival time was only 10 months for patients with EC transformation to SCC, among whom the longest survival (60 months) was achieved in a patient treated with triple-modality therapy comprising surgery, stereotactic radiosurgery, and chemotherapy (5). Similarly, a 2005 systematic review by Hamlat et al. of 52 patients with EC transformation to SCC demonstrated a median overall survival of 9 months (6). In our analysis of 59 cases, overall survival ranged from 1 day to 49 months with a median survival time of 6 months, and only two patients achieved survival exceeding 5 years. Importantly, modern reports from 2024 and 2025 utilizing advanced systemic options have largely confirmed these poor historical survival baselines rather than significantly prolonging them.

Prognosis is particularly dismal for patients presenting with LC, where the median survival is only 3 months, although adjuvant radiation therapy has been shown to extend median survival up to 26 months (6). In our case, the patient presented with LC and received adjuvant radiation therapy. She achieved a disease free survival of 17 months post re-operation, up until July 2026. Because early detection of malignant transformation and complete surgical clearance offer the best prospect for improved outcomes, long-term longitudinal surveillance- incorporating symptom monitoring, serial neuroimaging, and histological evaluation-is crucial for patients diagnosed with intracranial epidermoid cysts.

To better understand the mechanism of malignant transformation into SCC, identifying potential pathogenetic mechanisms as well as predictive, prognostic and therapeutic biomarkers is essential. Tsai et al. reported a programed death-ligand 1 (PD-L1; clone 22C3) tumor proportion score (TPS) of 40% (21), whereas Hanan et al. reported a PD-L1 TPS of 1% with a combined positive score (CPS) ≥ 1 (15). Additionally, amplifications in deubiquitinases, beta-defensins, alongside mutations in NOTCH2 and USP8 have been linked to immune evasion mechanisms, reflecting significant inter-tumoral heterogeneity and variable responses to immunotherapy (26). Comprehensive genomic profiling across reported cases has demonstrated microsatellite stability (MSS), loss of CDKN2A/B, MTAP (exons 2–8), and PTEN (exons 6–9) (27), as well as specific gene alterations including TP53 R158L, FANCM E684Q, FBXW7 Q492Vfs5, and RB1 S567 (15). Other identified genomic alterations include low tumor mutational burden (TMB), homozygous deletions in BARD1, MTAP and CDKN2A/B, a FANCA intron 10 mutation, an ETV6-CDK6 gene fusion and a TP53 R282P single nucleotide variant (21). Furthermore, Song et al. identified two pathogenic missense mutations: c.257C>T in the Gap junction protein beta 2 (GJB2) gene and c.1039A>G in the Toll-like receptor 2 (TLR2) gene (28). Personalized genomic profiling can help guide biology-driven targeted management, particularly for atypical presentations lacking standardized treatment protocols. Kondaboina et al. described molecular cross-talk between the PI3K-Akt-mTOR pathway, extracellular matrix (ECM) signaling and NOTCH2, highlighting potential therapeutic targets (26). However, because these molecular profiles originate from isolated case reports, definitive disease-wide commonalities remain to be established.

Although we conducted a comprehensive systematic search of all documented cases of intracranial epidermoid cyst transformation to SCC, the available literature consists entirely of isolated case reports characterized by significant treatment heterogeneity. Surgical approaches, extent of resection, radiation techniques and fractionation schedules, and systemic chemotherapeutic regimens varied widely across institutions yielding diverse survival outcomes and toxicity profiles. Consequently, pooled analysis of these historical cases cannot establish formal clinical practice guidelines or firm consensus recommendations. Specifically, while adjuvant radiotherapy has been shown to prolong survival, the optimal total radiation dose and fractionation scheme require further clinical validation.

## Limitations

Several methodological constraints must be considered when interpreting our findings. Primarily, the susceptibility to publication bias, as isolated case reports in neuro-oncology disproportionately feature atypical anatomic presentations or favorable treatment responses, potentially skewing pooled survival estimates upward. Additionally, our review spans over six decades (1960–2026). Over this period, diagnostic imaging evolved from cerebral angiography and early CT to high-field contrast-enhanced MRI, while radiation techniques shifted from 2D conventional fields to highly conformal IMRT, stereotactic radiosurgery, and proton therapy. Combining cases across these distinct eras introduces historical treatment heterogeneity that complicates direct comparisons.

Missing granular data in earlier literature also constrained our analysis. A substantial number of historical reports lacked detailed surgical descriptions to reliably classify resection extent (GTR vs. STR), omitted exact radiation dosimetry and fractionation protocols, or failed to record standardized follow-up intervals. Finally, because transformed intracranial SCC remains exceptionally rare, the small sample size (N = 59) and missing clinical variables limited our analysis to descriptive statistics and univariate comparisons, preventing multivariate Cox proportional hazards modeling to isolate independent prognostic factors.

## Conclusion

The malignant transformation of IECs into SCC is an exceedingly rare but severe complication with poor prognosis. Patients who have undergone resection of IECs require long-term surveillance to detect recurrence as well as potential malignant transformation. Radiological warning signs such as contrast enhancement, rapid growth and perilesional edema should prompt consideration of malignant change. Early detection, maximal safe surgical resection, and prompt adjuvant radiotherapy can provide the best prognosis. For patients unable to undergo complete resection, a multidisciplinary approach featuring neurosurgery, radiotherapy, and/or chemotherapy is recommended.
