# Supplementary material for: Malignant transformation of an intracranial epidermoid cyst into squamous cell carcinoma: a case report and systematic review
Source: Front Oncol. 2026 Oct 28;16:1978480. doi: 10.3389/fonc.2026.1978480 (PMC13751474; doi:10.3389/fonc.2026.1978480)
Supplement: Supplementary file 2 [file Supplementaryfile2.docx]

**Supplementary File 2**: Joanna Briggs Institute (JBI) Critical Appraisal Checklist for Case Reports

| **Study (Author, Year)** | **Q1** | **Q2** | **Q3** | **Q4** | **Q5** | **Q6** | **Q7** | **Q8** |
| --- | --- | --- | --- | --- | --- | --- | --- | --- |
| Davidson (1960) | Y | Y | Y | Y | Y | Y | U | Y |
| Fox H et al., (1965) | Y | Y | Y | Y | Y | Y | U | Y |
| Toglia (1965) | Y | Y | Y | Y | Y | Y | U | Y |
| Salyer (1973) | Y | Y | Y | Y | Y | Y | NA | Y |
| Garcia (1981) | Y | Y | Y | Y | Y | Y | Y | Y |
| Lewis (1983) | Y | Y | Y | Y | Y | Y | U | Y |
| Bondeson (1984) | Y | Y | Y | Y | Y | Y | NA | Y |
| Goldman S et al., 1987 | Y | Y | Y | Y | Y | Y | Y | Y |
| Abramson et al., 1989 | Y | Y | Y | Y | Y | Y | U | Y |
| Knorr (1991) | Y | Y | Y | Y | Y | Y | NA | Y |
| Delangre et al., (1992) | Y | Y | Y | Y | Y | U | U | Y |
| Kobayashi et al., 1993 | Y | Y | Y | Y | Y | Y | Y | Y |
| Acciarri et al., (1993) | Y | Y | Y | Y | Y | Y | NA | Y |
| Nishio et al., (1995) - Case 1 | Y | Y | Y | Y | Y | Y | Y | Y |
| Nishio et al., (1995) - Case 2 | Y | Y | Y | Y | Y | Y | U | Y |
| Fuse et al., (1995) | Y | Y | Y | Y | Y | Y | NA | Y |
| Bayindir et al., (1996) | Y | Y | Y | Y | Y | Y | U | Y |
| Murase et al., 1999 | Y | Y | Y | Y | Y | Y | Y | Y |
| Sawan et al., (2000) | Y | Y | Y | Y | Y | Y | U | Y |
| Tsugu et al., 2001 | Y | Y | Y | Y | Y | Y | Y | Y |
| Asahi et al., (2001) | Y | Y | Y | Y | Y | Y | NA | Y |
| Nawashiro et al., (2001) | Y | Y | Y | Y | Y | U | U | Y |
| Khan et al., (2001) | Y | Y | Y | Y | Y | Y | NA | Y |
| Link et al., (2002) | Y | Y | Y | Y | Y | Y | Y | Y |
| Monaco et al., (2003) | Y | Y | Y | Y | Y | Y | NA | Y |
| Hamlat et al.,(2004) (2003) | Y | Y | Y | Y | Y | Y | U | Y |
| Michael L et al., 2005 | Y | Y | Y | Y | Y | Y | Y | Y |
| Tamura K et al., 2006 | Y | Y | Y | Y | Y | Y | Y | Y |
| Ge P et al., 2009 | Y | Y | Y | Y | Y | U | U | Y |
| Kano T et al., 2010 | Y | Y | Y | Y | Y | Y | NA | Y |
| Nakao Y et al., 2010 | Y | Y | Y | Y | Y | Y | Y | Y |
| Hao S et al., 2010 | Y | Y | Y | Y | Y | Y | NA | Y |
| Lakhdar F et al 2011 | Y | Y | Y | Y | Y | Y | U | Y |
| Chon K et al., 2012 | Y | Y | Y | Y | Y | U | U | Y |
| Vellutini E et al., 2014 | Y | Y | Y | Y | Y | Y | NA | Y |
| Pikis S et al., 2016 | Y | Y | Y | Y | Y | Y | Y | Y |
| Ding S et al., 2016 | Y | Y | Y | Y | Y | Y | NA | Y |
| Seif B et al., 2017 | Y | Y | Y | Y | Y | U | U | Y |
| Mascarenhas A et al. (2017) | Y | Y | Y | Y | Y | Y | NA | Y |
| Roh et al (2017) | Y | Y | Y | Y | Y | Y | Y | Y |
| Liu et al., (2018) | Y | Y | Y | Y | Y | Y | NA | Y |
| Cuoco J et al 2019 | Y | Y | Y | Y | Y | Y | Y | Y |
| Kwon SM et al., 2019 - Case 1 | Y | Y | Y | Y | Y | Y | Y | Y |
| Kwon SM et al., 2019 - Case 2 | Y | Y | Y | Y | Y | Y | NA | Y |
| Chen et al., (2019) | Y | Y | Y | Y | Y | Y | Y | Y |
| Faltaous et al., (2019) | Y | Y | Y | Y | Y | Y | NA | Y |
| Zuo P et al., 2021 | Y | Y | Y | Y | Y | Y | NA | Y |
| Narasimhaiah et al. (2022) | Y | Y | Y | Y | Y | Y | Y | Y |
| Sakamoto et al., (2022) | Y | Y | Y | Y | Y | Y | Y | Y |
| Eatz T et al., 2023 | Y | Y | Y | Y | Y | Y | Y | Y |
| Zhang et al., (2023) | Y | Y | Y | Y | Y | Y | NA | Y |
| Jeon J et al., 2024 | Y | Y | Y | Y | Y | Y | Y | Y |
| Song et al., (2024) | Y | Y | Y | Y | Y | Y | Y | Y |
| Lin & Ding (2024) | Y | Y | Y | Y | Y | Y | NA | Y |
| Gasca Saldaña et al., 2025 | Y | Y | Y | Y | Y | Y | Y | Y |
| Alsadi et al., (2025) | Y | Y | Y | Y | Y | Y | Y | Y |
| Elsarraj & Hogan (2025) | Y | Y | Y | Y | Y | Y | Y | Y |
| Tsai C et al., 2026 | Y | Y | Y | Y | Y | Y | Y | Y |

**Assessment:**

Y = Yes, N = No, U = Unclear, NA = Not Applicable

1. **Q1:** Were patient's demographic characteristics clearly described?
2. **Q2:** Was the patient's history clearly described and presented as a timeline?
3. **Q3:** Was the current clinical condition of the patient on presentation clearly described?
4. **Q4:** Were diagnostic tests or assessment methods and the results clearly described?
5. **Q5:** Was the intervention(s) or treatment procedure(s) clearly described?
6. **Q6:** Was the post-intervention clinical condition clearly described?
7. **Q7:** Were adverse events (harms) or unanticipated events identified and described?
8. **Q8:** Does the case report provide takeaway lessons?
